# The dialogue as decision support; lived experiences of extended collaboration when an ambulance is called

**DOI:** 10.1080/17482631.2021.1970095

**Published:** 2021-08-24

**Authors:** Elin-Sofie Forsgärde, Anders Svensson, Mattias Rööst, Bengt Fridlund, Carina Elmqvist

**Affiliations:** a Department of Health and Caring Sciences, Linnaeus University, Växjö, Sweden; b Centre of Interprofessional Collaboration within Emergency Care CICE, Linnaeus University, Växjö, Sweden; c Ambulance Service, Region Kronoberg, Växjö, Sweden; d Department for Research and Development, Region Kronoberg, Växjö, Sweden; e Department of Clinical Sciences in Malmö, Family Medicine, Lund University, Malmö, Sweden

**Keywords:** Aged, emergency medical services, experiences of care, intersectoral collaboration, patients, prehospital emergency care, primary health care, reflective lifeworld research

## Abstract

**Purpose:**

This study aimed to describe extended collaboration in situations when an ambulance was called, as experienced by older patients, a significant other, and ambulance- and primary healthcare (PHC) centre personnel.

**Methods:**

The study used a phenomenological reflective lifeworld research (RLR) approach. Participants included in three specific situations with extended collaboration were interviewed: three older patients, one significant other, three ambulance personnel and four personnel at the PHC centre. The transcribed interviews were analysed for meanings of the phenomenon.

**Results:**

The extended collaboration means that decisions were supported through dialogue by bridging knowledge spaces between person, within-team and across-team levels. Through dialogue experience and knowledge were shared and certainty in decisions was increased. The extended collaboration was built on trust, responsibility taken, shared and entrusted, and the common goal of adapted care for the unique patient. A need for further improvement and transparency was elucidated.

**Conclusions:**

The difficulty of making care decisions stresses the importance of available extended collaboration based on the dialogue between patients, significant others, and ambulance- and PHC centre personnel to increase certainty in decisions. Collaboration further requires respectful encounters, trust, responsibility and a common goal of adapting the care for the unique patient.

## Introduction

There is an ongoing global discussion to meet the need of an ageing population by increasing collaboration between healthcare organizations in out-of-hospital settings (NHS, 2019; SOU 2020:19, 2020), such as between ambulance service and primary healthcare (PHC) centres (NHS, 2019). The need for ambulance care increases with age (Jones et al., 2017), and around 40–60% of patients engaging ambulance services are 65 years or older (Duong et al., 2018; Hjalmarsson et al., 2020). There is an upward trend for older patients to receive their entire care in out-of-hospital settings, such as in their own home or at PHC centres after calling for an ambulance (Forsgärde et al., 2020; Hjalmarsson et al., 2020). Not being transported by an ambulance to the emergency department (ED) is also known as non-conveyance to hospitals. Patients with ambulatory care sensitive conditions, meaning conditions that can be treated in out-of-hospital settings, receive optimal care through, e.g., timely PHC (Conley et al., 2016). Care level decisions, however, include risks where inadequate decisions harm patients (Ackroyd-Stolarz et al., 2011; Ebben et al., 2017). Patients perceive optimal care as available, with continuity and adjusted to their needs (Bayliss et al., 2008). The decision to call for an ambulance is taken acutely by older patients or significant others (Van der Kluit et al., 2018). Factors leading to the decision are situations that cannot be self-handled and when no other choice exists (Booker et al., 2017; Van der Kluit et al., 2018). Older patients do not discuss their wishes of care levels with the significant other before calling for an ambulance (Van der Kluit et al., 2018). Older patients’ and significant others’ perceptions of optimal care levels are diverse. Some older patients experience safety when obtaining care at the ED despite the busy, chaotic and demanding experience (Arendts et al., 2015); others want to avoid the ED due to earlier experience of non-caring encounters (Smith-Carrier et al., 2017). Patients’ perception of optimal care level is, however, affected by ambulance personnel assessment, and the responsibility for the care level decision often handed over to the ambulance personnel (Booker et al., 2017). Patients experience confidence in ambulance personnel decisions of non-conveyance to hospitals when getting self-care instructions or receiving an appointment to their specialist in general practice (GP) (Van Doorn et al., 2021). Significant others and healthcare professionals experience optimal care levels for older patients when care is delivered at home due to the risk of disorientation, distress, lack of basic care and incorrect medication during ED visits (Arendts et al., 2015; Goodridge & Stempien, 2019).

Care level decisions are challenging for ambulance personnel to make and are influenced by multiple factors, both patient-related and healthcare system-related (Ebben et al., 2017). Ambulance personnel’s experiences of decisions of non-conveyance to hospitals are diverse, including a sense of certainty, fear and uncertainty, and doubting the correctness of the decision (Backman et al., 2019; Lederman et al., 2019). The diverse experiences are possibly connected to the degree of complexity of patients’ symptoms. Older patients have more complex symptom-pictures than younger patients due to age-related deteriorations in physiological responses that increases risk of diseases and the need for pharmacies (Lipsitz, 2002). Collaboration between ambulance personnel and GPs at PHC centres increases non-conveyance to hospital decisions (Larsson et al., 2017; Villarreal et al., 2017), without increased risk for patients (Larsson et al., 2017). GPs support around 8% of ambulance calls in the United Kingdoms (UK) either through telephone or through assessment at PHC centres, mostly involving patients with musculoskeletal or neurological symptoms (Villarreal et al., 2017). In Sweden, GPs assess 2% of patients calling for an ambulance at PHC centres (Forsgärde et al., 2020). GPs at PHC centres are skilled in assessing and making decisions when complexity and uncertainty exist (Villarreal et al., 2017). However, there is limited knowledge about how patients, significant others, and healthcare professionals experience collaboration between ambulance personnel and personnel at PHC centres. Previous studies show diverging results identifying both positive experiences (Villarreal et al., 2017) and collaborative difficulties (Forland et al., 2009).

## Aim

This study aims to describe extended collaboration in situations when an ambulance is called, as it is experienced by older patients, a significant other, and ambulance- and PHC centre personnel.

## Method

### Design

This study had a descriptive design with a reflective lifeworld research (RLR) approach founded on the continental philosophy of phenomenology (Dahlberg & Dahlberg, 2019; Dahlberg et al., 2008). The goal of the RLR approach was to describe the meanings of the phenomenon, namely extended collaboration when an ambulance is called. RLR’s methodological principles of openness, flexibility and bridling were conducted during the whole research process: being open and flexible towards the phenomenon and at the same time bridling the understanding by adopting a reflective attitude, meaning holding back and reflecting on the understanding, to avoid jumping to conclusions (Dahlberg & Dahlberg, 2019; Dahlberg et al., 2008).

### Setting

The study took place in a municipality in the southern part of Sweden. Sweden has public-provided health care and accessibility to telephone advice nurses giving patients care advice and to the emergency medical communication centre (EMCC) dispatching ambulances around the clock. The municipality area was approximately 980 km^2^ and included 20,000 inhabitants, where 22% were ≥ 65 years old (Statistics of Alvesta Municipality, 2018). Furthermore, the area held one ambulance station and three PHC centres, only one of which was included in the study. The ambulance station had one ambulance around the clock and another during the daytime (Monday-Friday). The ambulances were mostly staffed, with RNs and specialist ambulance nurses (SANs). SANs were RNs with an additional combined ambulance care vocational programme and master’s programme (Sjölin et al., 2015). The ambulances were further staffed with a limited number of emergency medical technicians (EMTs). The combination of RNs, SANs and occasionally EMTs are common in the Swedish ambulance service. In this study, all the previously described professions are referred to as ambulance personnel. The ambulance personnel in this region worked in a team of two, including a minimum of one SAN. The ambulance service shared the electronic health record with PHC centres. Inhabitants in Sweden choose their PHC centre, and the studied PHC centre had 11,000 listed patients. The PHC centre was open during the day-time (Monday-Friday) and staffed by associated nurses, RNs and GPs with the nearest hospital located within 20 kilometres. At the PHC centre, one RN was responsible for receiving calls from ambulance personnel, and one GP was responsible for being available to support all RNs at the PHC centre and assessing patients coming to the PHC centre by ambulance. In this study, when personnel from both the ambulance services and the PHC centre are being referred to, they are called healthcare professionals.

### Extended collaboration

The extended collaboration was developed through a common guideline between the ambulance service and the PHC centre. The guideline goal was to offer patients calling for an ambulance an alternative care level setting apart from the ED. A routine was established where the ambulance personnel were enabled to consult an RN and GP at the PHC centre. The guideline included patients triaged with non-life-threatening conditions (yellow, green or blue) according to the Swedish five-grade triage system: Rapid emergency triage and treatment system (RETTS©) (Widgren, 2012).

**Figure 1.**
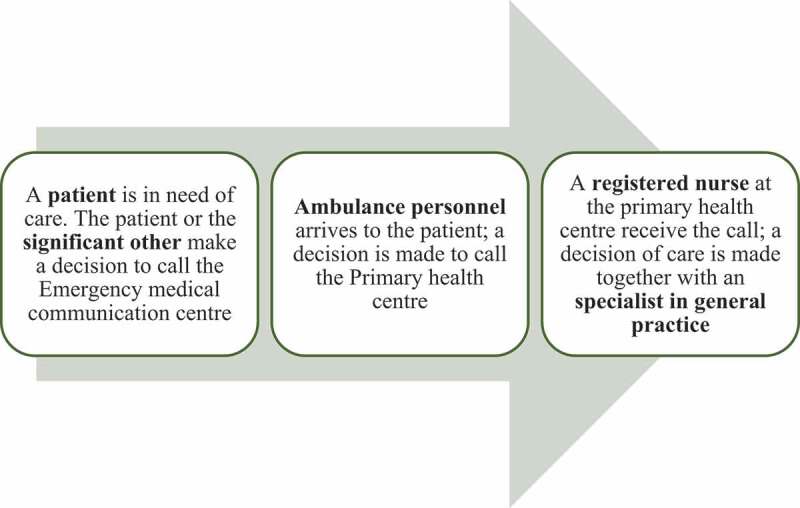
An overview of when the extended collaboration started for the patient, the significant other, ambulance personnel and personnel at the primary health centre

For patients, the extended collaboration situation started with the decision to call the EMCC for an ambulance. For the ambulance personnel, the extended collaborative healthcare situation started onsite when making a decision according to the guideline of contacting RNs at the PHC centre. For the personnel at the PHC centre, the extended collaboration situation started with a phone call from the ambulance personnel (Figure 1). At the PHC centre, the RN and GP further assessed the patient, and the GP decided whether patients would receive optimal care at the PHC centre or the ED.

### Situations and participants

In this study, the extended collaboration led to one of three different situations, namely, 1) A patient was after the ambulance personnel assessment waiting within the own home for a follow-up by a GP at the PHC the same day, 2) A patient was after the ambulance personnel assessment transported to the PHC, 3) A patient was after the ambulance personnel assessment transported to the PHC, and after the GP assessment at the PHC further transported by ambulance to the ED (Table I).

**Table I.** An overview of the three studied situations, participant inclusion and variation during extended collaboration when an ambulance is called

Situation	1	2	3
Description	A patient received assessment by ambulance personnel within the own home and awaited follow-up by a specialist in general practice at the primary healthcare centre the same day	A patient received assessment within the own home by ambulance personnel and was thereafter transported by an ambulance to the primary healthcare centre	A patient received assessment at an accident site by ambulance personnel and was thereafter transported by an ambulance to the primary healthcare centre, followed by an assessment by a specialist in general practice, and was thereafter transported by ambulance to the emergency department
Patient 65–79 years	Female	-	-
Patient > 80 years	-	Female	Female
Symptom/Cause	Chest pain	Shoulder pain	Bike accident
Significant other	-	Daughter	-
Registered nurse, Ambulance service	-	-	Male
Specialist ambulance nurse Ambulance service	Female	Male	-
Registered nurse at the primary health care centre	Female	-	Female
Specialist in general practice at the primary healthcare centre	-	Female	Female

To receive a comprehensive picture of the studied phenomenon, the inclusion criteria were patients ≥ 65 years old, significant other and healthcare professionals included in the three situations. All situation descriptions included the majority of participants involved. The healthcare professionals included were ambulance personnel, i.e., RN and SAN, and personnel at the PHC centre, i.e., RN and GP. With the exception of situations, the intention was also to include variations in participants: patient age, symptom and gender and healthcare professionals’ role and gender. Patient age was divided according to the WHO’s definition of older age, namely 65–79 years (younger older) and > 80 years (older) (World Health Organization, WHO, 2001). The exclusion criteria were patients with difficulty of expression due to decreased cognitive function without a significant other supporting them during the interview. Eleven participants were finally included in the study: three patients, one significant other, three ambulance personnel and four personnel at the PHC centre.

### Data collection

Two contact-persons, the ambulance service IT manager and an RN working at the PHC centre, identified situations according to the inclusion criteria. When a situation was identified, the first author was informed without revealing sensitive patient information. The contact-person then sent study information letters to the patient and healthcare professionals included in the situation, who in turn contacted the first author by letter with a consensus of participation and were then contacted by the first author.

The individual interviews, i.e., the participants’ description of their lived experiences of the phenomenon, were performed between October 2017 and December 2018. The interviews were conducted in a setting chosen by the participant, at their home or their place of work. One participant had a significant other during the interview for memory support. The significant other was thereafter interviewed individually by telephone. Before the interview started, the participants were informed about which situation being studied. In line with the RLR approach, the interviews started with one initial open-ended question for all participants in relation to the phenomenon: “Can you please describe your experience of the actual situation”. The follow-up questions, “you mention that … can you describe more?” and, “How was that?”, were aimed to obtain in-depth information of their lived experience of the phenomenon. The interviews lasted for 35–70 min and were transcribed verbatim by the first author.

### Data analysis

The analysis was conducted through repeated movement between the whole material, the parts, and the new whole to validate the accuracy of the analysis and describe the phenomenon’s essence, i.e., the meaning structure (Dahlberg et al., 2008). Firstly, the transcribed interviews from each situation, namely the situation description, were read several times isolated from the other situation descriptions to understand the diversity and the wholeness of the studied phenomenon (Elmqvist et al., 2008). Subsequently, meaning units, e.g., sentences or parts of the text connected to the phenomenon were extracted from the interview text, and meanings within each unit were identified. Each meaning unit was looked upon as a figure with the other meaning units as background to support understanding. Similar meanings within each situation description were grouped into patterns (Dahlberg et al., 2008). Further, similarities and differences between patterns from all situation descriptions were identified by relating them to each other and similar patterns were grouped and abstracted into clusters. The phenomenon’s essence was identified by relating clusters to each other to find invariant meanings existing in between clusters, i.e., the constant meanings describing the essence of the phenomenon. The constituents further describe variations of the essence (Dahlberg et al., 2008). The first and last author conducted regular dialogues at the beginning of the analysis process and with all authors during the result description. The analysis was further reviewed at three seminaries by other researchers and doctoral students with practical experience of caring for older patients as ambulance personnel or RNs in hospital care. The results firstly describe the phenomenon’s essence, followed by its constituents. Quotations are included to contextualize and to strengthen the result.

## Ethical considerations

The study was approved by the ambulance service, the PHC centre and the Swedish Ethical Review Authority, Dnr 2017/348-31. The participants were assured through the information letter and verbally prior to the interview that their participation was handled with confidentiality, that it was voluntary to participate without impact on future care, and that they could withdraw at any time according to the Helsinki declaration (World Medical Association, WMA, 2013).

## Results

### Essence

The extended collaboration when an ambulance is called bridges knowledge spaces through dialogue within and in-between the triad of levels at person, within-team and across-team levels as decision support. The situation starts with a comprehensive estimation of unique needs and the possibility of fulfiling them, where uncertainty and certainty coexist. Previous experience and knowledge give on one hand the certainty that care is needed. On the other hand, there is uncertainty and a need for confirmation of assessment and decisions. The fact of a right or wrong decision is lacking, and support is searched for and gained through dialogue in which experience and knowledge are shared. A common comprehensive understanding is developed within the dialogue and this gives certainty in decisions. Through dialogue as decision support, responsibility for the care is taken, shared and entrusted. Trust is the core of entrusting and receiving responsibility. Extended collaboration is possible due to a common goal; adapted care, and when achievable, giving available and time-saving care, are still in need of improvement and transparency.

### A movement towards increased certainty

Extended collaboration when an ambulance is called increases certainty in decisions for the patient, the significant other and the healthcare professional. A movement towards increased certainty begins with experiences, knowledge and perceived severity, providing the patient and the significant other with certainty that care is needed. At the same time, uncertainty exists about the cause of the symptoms. The uncertainty of calling EMCC for an ambulance is based on concerns that own decision is incorrect, with a risk of being accused of not being sufficiently ill or having diffuse symptoms being trivialized or misinterpreted. Fear of severe illness offers, however, no choice but to call.

*“Well, you actually get scared when something happens, and you think … what do I do now? Can I take an ambulance just for that?”* Patient B.

Increased certainty in the decision to call the EMCC for an ambulance occurs when ambulance personnel confirm to the patient and the significant other that the decision was correct. In the care encounter, fear of life-threatening conditions decreases, but existential anxiety does not disappear until the symptoms are relieved.

To be told that it is okay to call (the ambulance) … it gives you confidence … “you are so welcome to call when you need us” … then you think that I can call the ambulance when I’m not feeling well Patient A.

The situations begin with certainty within the professional role coexisting to a different extent with an uncertainty of making adapted decisions for the unique patient. Advance information from EMCC or ambulance personnel about a patient’s condition provides preparation for the healthcare professional. Simultaneously, there is uncertainty about whether the information is perceived correctly and thereby weighed into the comprehensive assessment. Previous relational knowledge about the patient makes it possible to compare the current condition with the patient’s habitual situation, which shortens the time of the healthcare professional’s decision. The comprehensive assessment of the unique patient, on one hand, provides increased certainty in the decision about the level of care. On the other hand, the certainty is affected by the knowledge that serious illness can exist without deviating findings and can quickly deteriorate the patient’s condition.

… Sometimes, not with simple symptoms, but the other ones, when you don’t really know which decision to make … then I think “Do I think right?” … ” Is this an orthopaedical or medical cause”? Ambulance personnel C.

Certainty in decisions about care levels arises when the healthcare professional are confirmed in the decision by the patient, the significant other and other healthcare professionals. When confirmation lacks, the ambulance personnel follow up the decision through the patient medical record, and the GP receives feedback on decisions through follow-up conversations with the patient.

… then you describe the dilemma, and they (personnel at the PHC) say “yes, we will manage this” … It feels great because it confirms your decision. Ambulance personnel B.

### The dialogue as decision support

Extended collaboration when an ambulance is called means that decision support is searched for and gained through dialogue. To avoid being alone in decisions of calling EMCC for an ambulance, the patient searches for support through dialogue with significant others and healthcare professionals. Through the dialogue, the patient and significant other give information about the situation to ensure that the healthcare professional’s decisions are based on the correct information, and they receive information from the healthcare professional’s assessment.

So I mean, sometimes you think, ‘How can I explain myself now so I know I’m getting it right’ Because it has happened that they (healthcare professional) interpreted it (the explained symptoms) as something else. Patient B.

Moreover, through the dialogue, the patient and significant other are invited to be involved, assess the reasonableness, and approve and influence care level decisions. The information mediated within the dialogue adds to a common comprehensive understanding between the patient, the significant other and the healthcare professional, and this supports decisions. A lack of dialogue restrains a common comprehensive understanding, and thereby patients’ and significant others’ support.

… then they (Ambulance personnel) made an assessment that she rather get care at the PHC, and I said it was okay. I saw that it was not as bad as it usually is when we go to the hospital. Significant other A.

In the dialogue, the healthcare professional support the patient to tell their story to grasp an understanding of the situation. Through dialogue, decision support is searched for by the ambulance personnel with all involved when decision tools are lacking or do not provide complete support, due to being designed too generally or not congruently with the structural assessment.

It is important to come to an agreement … it has to be an interaction between the patient and you. Because if you do not have them with you, it will not work. Ambulance personnel B.

The limited treatment possibilities at PHC centres and generally-designed guidelines, however, provide support for the GP’s decisions of what can be managed at the PHC centre. The GP further searches for support through dialogue with the patient, significant other and the RN at the PHC centre to adapt the decision to the unique patient. Through the dialogue, experiences, knowledge and assessment result are shared between all involved and are gathered into a common comprehensive understanding, a decision support that increases the surety of making the right decisions.

So I mean, just this to make assessments … sometimes it is very simple, it is so clear, and sometimes it is more diffuse … and then we work as in dialogue with ambulance personnel and with the GP … or whoever that is involved, I think it’s quite enriching … RN at the PHC centre B.

### A strive for trust and shared responsibility

Extended collaboration when an ambulance is called means that trust is needed to share responsibility for the care. On one hand, at the beginning of the care encounter, trust exists in the patient and significant other’s own and others’ knowledge and intentions to help. On the other hand, previous negative care encounters increase uncertainty and distrust. With a wish to entrust or share responsibility for decisions and care with healthcare professionals, their competence and intention to help are judged, and trust is valued by the patient and the significant other. Trust is developed when being treated with respect and receiving a comprehensive assessment based on the unique situation.

You feel quite small when you come in (to the PHC) … then they (healthcare professional) are so kind and they do exactly what they can and I am so grateful … Patient C.

When trust is developed, the patient entrusts or shares responsibility with significant others and the healthcare professional for the decision of adapted care. The trust remains in the next encounter with the same healthcare professional. The significant other only partly entrusts the responsibility for the patient’s care to the healthcare professional by not letting go of evaluating and monitoring changes in the patient’s condition.

- I think that when you are down, then you don’t care, I can sit and just fall asleep (patient). - If you have a relative with you, then you don’t care where you are, Mother (significant other). - Yes, I know that someone is there (patient). - I think it would have been worse if you had been left alone (significant other). - Well then I would have been more tensed and felt that I have to do this and that and remember things (patient). Patient B and significant other A.

Trust develops within respectful care encounters and provides a sound basis for further care encounters with the healthcare professional. Responsibility is taken by the healthcare professional to gain trust from the patient and the significant others in decisions of an adapted care level.

… if you come in with an acute illness, you are more exposed, you are more worried … and that relationship can either go very wrong, and you can really lose trust … but often you gain trust … I feel that these situations can create stronger bonds to the patient that last in the future … GP at the PHC centre A.

Simultaneously, extended collaboration is built on established trust and shared responsibility between ambulance personnel and personnel at the PHC centre due to previous respectful and welcoming encounters.

And what I experience is good in comparison to other PHCs … where ambulance personnel could question my decisions, I have never experienced that here … GP at the PHC centre B.

The common trust is further grounded on the idea that the knowledge from all involved and the enlarged assessment at the PHC centre improves the decision ground of finding a care level adapted to the patient’s needs. To not misuse the trust, the ambulance personnel entrust the responsibility for the patient’s care to personnel at PHC centre only when they consider the patient’s symptoms to be relevant for the PHC centre. Responsibility for further assessment and decisions is taken by the personnel at the PHC centre.

### A possibility or limitation for adapted care

Extended collaboration when an ambulance is called enables or limits the common goal of adapting care for the unique patient’s needs. On one hand, the care expectations are exceeded for the patient and the significant other due to the accessible and time-saving care path. The received care meets the desire for an adapted and de-dramatized care.

It depends; if I feel that I am getting better, I prefer to be at home, but when I feel that it is bad and that I cannot handle it, then I go with them (the ambulance personnel) Patient A.

The PHC centre provides a calm, well-known environment with the healthcare professional previously talked to or met, making the patient feel safe. On the other hand, PHC centres’ opening hours limits possibilities for adapted care. Lack of information from the personnel at the PHC centre during the visit further limits the patient’s and the significant other’s inclusion in the care.

So we ended up getting a cortisone injection and had to sit for an hour and wait for the car that was going to take us to the X-ray. That waiting time was almost the worst. Significant other A.

The extended collaboration, however, gives the patient and the significant other hope for additional opportunities for adapted care, with a desire for healthcare professional other than ambulance personnel to be available for assessment in patients’ homes to decrease uncertainty when calling for help.

Extended collaboration provides, on one hand, possibilities for an available care path to reach healthcare professionals’ common goal of adapting care for the patient’s needs, where patients with less severe symptoms meet the conditions to be cared for at home or the PHC centre.

… It is a huge advantage (the extended collaboration), especially for the patient … this lady got to come to the PHC, where she wanted … she did not need to go to the ED, and for us, it is a huge gain and security to have the opportunity … Ambulance personnel A.

The willingness to be the first instance providing an extended comprehensive assessment of the patient stimulates and enriches the personnel at the PHC centre. Extended collaboration is possible due to the PHC centres’ organization promoting patients from ambulances, even though the knowledge of the extended collaboration guideline is not widely spread among personnel at the PHC centre.

No, I have never seen the extended collaboration guideline. I have also never experienced collaboration difficulties with the ambulance personnel RN at the PHC centre A.

On the other hand, extended collaboration is limited by time factors and affected negatively by increasing workload and lack of GPs. The limitation further affects the common desire for future additional development of care adapted for the unique patient.

… The workload for the GP has become much heavier lately because you suddenly get a lot more patients and then it can get too tight when patients from the ambulance come as well. GP at the PHC centre B.

## Discussion

Extended collaboration when an ambulance is called means that decisions are supported through dialogue. Dialogue bridges the knowledge spaces within and in between the triad of levels: person level (patients and significant others), within-team level (healthcare professionals in the same organization), and across-team level (in between ambulance personnel and personnel at the PHC centre). The support consists of a common comprehensive understanding developed through experience- and knowledge-sharing that increases certainty in decisions. Extended collaboration is based on trust, and that responsibility is taken, shared and entrusted. Collaboration is possible due to the common goal; adapted care based on patients’ unique needs.

Consequently, the discussion focuses on uncertainty, dialogue and the goal of adapted care with implementations for the triad of levels. In this study, uncertainty is experienced to different extent within the triad of levels and is the starting point for extended collaboration to increase certainty in decisions. No previous studies to our knowledge describe increased certainty during collaboration in between the specific triad of levels. Previous studies, however, show similar results for the existence of uncertainty for patients (Holmberg et al., 2014), significant others (Rantala et al., 2015), ambulance personnel (Booker et al., 2017) and GPs (Stolper et al., 2021). At the beginning of a care process, there is a high degree of uncertainty due to limited knowledge of a patient’s condition (Breitsameter, 2010; Croskerry, 2002). The uncertainty is connected to the responsibility and risk of making wrong decisions (Wallinvirta, 2011) and is experienced by healthcare professionals as burdensome with feelings of guilt, insufficiency and concerns (Strandberg & Jansson, 2003). Risks are beyond personal control and are present in complex situations where decisions are to be made with incomplete information (Breitsameter, 2010). Decisions of non-conveyance are burdensome for ambulance personnel due to the embedment of the responsibility and risk of making wrong decisions compared to the decision to convey patients to the ED (Lederman et al., 2019; O’Hara et al., 2015). The responsibility for making non-conveyance decisions is questioned by ambulance personnel (Lederman et al., 2019). Both willingness and ability in form of knowledge, support and available instances are needed when responsibility for adapted care is to be taken (Wallinvirta, 2011). When ambulance personnel entrust and share responsibility for patient care with GPs, they are not alone responsible for making a decision of non-conveyance; entrusting responsibility to GPs can be interpreted as an act of taking responsibility for adapting the care for patients (Larsson et al., 2017; Villarreal et al., 2017). The certainty in decisions increases with added knowledge through, e.g., structural assessment, history taking and diagnostics (Breitsameter, 2010; Croskerry, 2002). The extended collaboration within this study offers increased knowledge and certainty through the extended assessment at PHC centres. The fact that uncertainty always exists to some extent in the beginning of a care process points out that strategies should be focused on limiting uncertainty in different ways (Breitsameter, 2010). At a person level, the result highlights the need for respectful encounters, including confirming with patients and significant others the difficulty of knowing where to call when in need of healthcare. Within-team and across-team levels, there is a need for available and permissive collaborative support between ambulance personnel and personnel at the PHC centre as a strategy to limit uncertainty during care level decisions in out-of-hospital settings.

In this study, decision support is gained through dialogue within and in between the triad of levels, and the shared and entrusted responsibility within the extended collaboration is based on trust. Previous studies’ result show increased rates of non-conveyance to hospitals during the collaboration between ambulance personnel and GPs, indicating that support in decisions is gained through the specific collaboration (Larsson et al., 2017; Villarreal et al., 2017). A deeper description of experiences of the decision support through the specific collaboration however lacks. Other studies found a similar result of dialogue being important to establish trust (Norberg Boysen et al., 2017), and that patients cared for in PHC centres and EDs when calling for an ambulance have similar trust in the received care (Norberg Boysen et al., 2019). There is always an understanding gap in the dialogue due to encounters consisting of persons with different lifeworlds, meaning that previous experiences and knowledge affect the understanding of the situation (Dahlberg et al., 2008). In this study, the dialogue bridges the knowledge spaces within and in between the triad of levels when experiences and knowledge are shared, entailing a common comprehensive understanding that increases the knowledge ground and gives support to decisions. In dialogue, responsibility is taken when adding experience, knowledge and critical thinking into a shared decision that decreases biases (Mercier & Sperber, 2017). All those involved in shared decisions become part of a safety system that identifies risks and arguments for adapted care (Varpio et al., 2008). On the contrary, if responsibility is not taken within collaborations group-thinking emerges that creates a diffusion of responsibility, e.g., no one takes responsibility for the decision. Group-thinking leads to limited options and plausible unethical group decisions (Mannion & Thompson, 2014). During an ethical shared decision, the whole triad of levels is included in the dialogue and has the same value when decisions are made (Breitsameter, 2010). Patients and significant others become involved and empowered through the dialogue with healthcare professionals. Dialogue difficulties, ignorance, and disrespectful encounters hinder the involvement and make patients and significant others feel excluded and powerless (Rantala et al., 2015, 2016). Patients and significant others need clear and understandable information to make decisions, which requires healthcare professionals to use cognitive and emotional competence when informing about decision grounds, possible consequences, and side effects of different options (Breitsameter, 2010). At person, within-team and across-team levels the result highlights the importance of dialogue as decision support and involvement of the triad of levels when making decisions about adapted care for the unique patient in complex situations.

In this study, extended collaboration was possible due to the triad of levels common goal of adapting the care level decision to the unique patient. A common goal has been pointed out as the key factor of good collaboration (Breitsameter, 2010; Sodomin, 2016). Patients in the study had no specific goal of visiting the ED, but rather to be released from their symptoms. Care fails if patients’ interests and needs are subordinated to the interests of healthcare organizations (Breitsameter, 2010). Sweden lacks a national care level decision support within ambulance services (Lederman et al., 2018). Such decision support has the prerequisite to strengthen confidence during decision-making but not fully meet the needs for individual adaption in complex situations (Croskerry, 2002). Decisions are not only based on medical severity; to gain a comprehensive understanding of the unique patients’ needs, the ambulance personnel further add information of patients’ lifeworld, social support and living conditions (Holmberg, 2021). To adapt a care level decision to the unique patient, means to sometimes go outside recommendations of guidelines on why healthcare professionals need support in making adequate decisions from their organization (Lederman et al., 2019). GPs’ medical knowledge and relational knowledge of the patient add to the comprehensive understanding and broaden the decision ground, which reduces hospital visits for patients with ambulatory care sensitive conditions (Barker et al., 2017). However, PHC centres sufficiently staffed by GPs are a prerequisite to enable continuity of care that is the basis of relational knowledge and to improve collaborative care in out-of-hospital settings (SOU 2020:19, 2020). The result highlights the benefits of an extended dialogue-based collaboration for the triad of levels and its possibilities of being a future strategy to ensure optimal care level for individual patients. For this to be effective there is a need to focus on the importance of ensuring continuity in PHC.

### Methodological considerations

Methodological quality was reached by following the RLR methodological principles at all stages in the research process (Dahlberg et al., 2008; Van Wijngaarden et al., 2017). The intention was to have variations among the participants to broaden the description of the phenomenon. A possible limitation was that the majority of the participants were females, but a strength was the rich descriptions from each participant. Validity was maintained due to the participants all having lived experience of the studied phenomenon, and the result focused on meanings structure of the phenomenon instead of participants’ experiences (Van Wijngaarden et al., 2017). The inclusion of meanings from all participants’ lived experiences in all situations deepened the understanding of the phenomenon. Objectivity was gained through reflections and curiosity, with the authors’ remaining open and flexible towards the phenomenon by bridling the understanding to not understand the phenomenon too quickly (Van Wijngaarden et al., 2017). Regular dialogues between the authors and discussions with peers helped keep a distance from the phenomenon and discover what was not yet known. Further, the structured analysis of going from the parts to the whole several times, making sure that the meanings were well-grounded within the participants’ lived experiences. The phenomenon’s abstracted essence makes it possible to transfer the result from the specific participant within the study to similar settings and conditions (Dahlberg et al., 2008; Van Wijngaarden et al., 2017).

## Conclusions and implications

The results show that extended collaboration when an ambulance is called gives support in decisions through dialogue. Dialogue increases certainty through experience- and knowledge-sharing between all involved. Furthermore, dialogue bridges knowledge spaces between person, within-team, and across-team levels into a common comprehensive understanding of patients’ needs. The extended collaboration is further based on trust, responsibility and the common goal of adapting care to the unique patient.

The result highlights the importance of available and permissive dialogue-based collaborative support between patient, significant other, ambulance personnel and personnel at the PHC centre during decisions in complex situations. At a person level, respectful encounters are required to confirm the difficulties of deciding to call for an ambulance. At person, within team and across-team levels, the inclusion of all parties in the dialogue extends the decision ground and increases certainty in decisions. For extended collaborations, trust, responsibility and a common goal of adapting the care for the unique patient are required. Finally, continuity in primary care needs to be improved to maintain and extend collaborative care in out-of-hospital settings. Further research is needed to explore optimal urgent care for older patients in out-of-hospital settings.
